# Demand Ischemia as a Predictor of Mortality in Older Patients With Delirium

**DOI:** 10.3389/fcvm.2022.917252

**Published:** 2022-06-06

**Authors:** Manish Kumar, Shivaraj Patil, Lucas Da Cunha Godoy, Chia-Ling Kuo, Helen Swede, George A. Kuchel, Kai Chen

**Affiliations:** ^1^Department of Medicine, Pat and Jim Calhoun Cardiology Center, University of Connecticut, Farmington, CT, United States; ^2^Department of Internal Medicine, University of Connecticut, Farmington, CT, United States; ^3^Connecticut Convergence Institute for Translation in Regenerative Engineering, University of Connecticut Health, Farmington, CT, United States; ^4^Department of Public Health Sciences, University of Connecticut School of Medicine, Farmington, CT, United States; ^5^UConn Center of Aging, University of Connecticut School of Medicine, Farmington, CT, United States

**Keywords:** delirium, demand ischemia, mortality, coronary artery disease, clinical outcomes

## Abstract

**Introduction:**

The impact of demand ischemia on clinical outcomes in patients with delirium remains largely unexplored. This study aims to evaluate the effects of demand ischemia in older patients with delirium on in-hospital mortality and length of stay (LOS) using the largest US inpatient care database, National Inpatient Sample (NIS).

**Methods:**

We obtained data from the year 2010 to 2014 National Inpatient Sample (NIS). We used the International Classification of Diseases-Ninth Edition-Clinical Modification (ICD-9-CM) diagnosis codes to identify all the records with a primary or secondary diagnosis of delirium with or without demand ischemia and other clinical characteristics. We then compared in-hospital mortality and length of stay (LOS) in patients with and without demand ischemia.

**Results:**

We analyzed 232,137 records. Patients with demand ischemia had higher overall in-hospital mortality than those without demand ischemia (28 vs. 12%, *p* < 0.001). After adjusting for clinical comorbidities and complications, demand ischemia was no longer associated with increased in-hospital mortality (OR: 1.14; 95% CI: 0.96–1.35; *p* = 0.141). However, further analysis with the exclusion of critically ill patients with non-cardiogenic shock or mechanical ventilation showed a significant association of demand ischemia with increased in-hospital mortality (adjusted OR: 1.39; 95% CI: 1.13–1.71; *p* = 0.002). Among non-critically ill survivors, patients with demand ischemia had a longer median LOS [4, (3–7) days] than those without demand ischemia [4, (2–6) days] (*p* < 0.001). However, the difference was not statistically significant after adjustment for covariates.

**Conclusion/Relevance:**

Demand ischemia did not affect mortality in critically sick patients. In non-critically ill patients, however, demand ischemia was significantly associated with increased in-hospital mortality, likely due to the severity of the underlying acute illness. Measures aimed at mitigating risk factors that contribute to delirium and/or demand ischemia need to be explored.

## Introduction

Cardiovascular disease (CVD) is the leading cause of death in the United States and worldwide, inflicting a significant societal burden in terms of morbidity, mortality, disability, and healthcare costs (1). Coronary artery disease (CAD) is one of the most common CVDs seen in older adults. The incidence and prevalence of CAD increase with age (2). CAD often presents as an acute coronary syndrome (ACS) wherein a plaque rupture or erosion causes total or subtotal coronary artery obstruction and subsequent myocardial ischemia/necrosis (Type 1 myocardial infarction, T1MI). This myocardial injury causes elevation of cardiac biomarkers such as cardiac troponin T and troponin I (3). Troponin assays detect minor myocardial injury with high sensitivity and specificity and have become the cornerstone for the diagnosis of myocardial injury. These cardiac biomarkers, however, do not define the cause of myocardial injury and can be elevated in the absence of coronary artery obstruction and non-coronary diseases as well (4). This non-coronary release of troponin occurs due to a mismatch between myocardial oxygen demand and supply and is known as demand ischemia or Type II myocardial infarction (T2MI) (3, 4). The hallmark of type II MI is the absence of a plaque rupture or erosion as the primary cause of this release and can occur both in the presence or absence of obstructive CAD. This may happen in several non-thrombotic coronary and systemic diseases such as sepsis, hypoxia, anemia, arrhythmia, congestive heart failure, pulmonary embolism, myocarditis, and renal failure (4).

One such condition that may elicit an imbalance between myocardial oxygen demand and supply is delirium. Delirium is a neuropsychiatric syndrome frequently encountered in hospitalized old patients. The reported prevalence of delirium varies by patient group and setting and ranges from 23% in medical inpatients to 83% in the intensive care unit (5, 6). Delirium results in an altered state of arousal ranging from hypoactive response to hypervigilance, psychosis, and severe agitation. The neuropsychiatric symptoms cause severe physiological distress with subsequent catecholamine release, which in turn, can invoke demand ischemia (7, 8). Of note, both ischemia and delirium not only coexist in hospitalized older patients but also share similar precipitating factors and pathophysiological mechanisms (4, 5). Thus, it is of clinical importance to evaluate the impact of myocardial infarction on outcomes in the setting of delirium. In patients with T1MI, the occurrence of delirium is associated with increased mortality (9, 10). However, the impact of T2MI, and delirium on clinical outcomes is largely unexplored. In this study, we aim to evaluate the effects of demand ischemia in hospitalized older patients with delirium on in-hospital mortality and length of stay (LOS) using the largest US inpatient care database, National Inpatient Sample (NIS).

## Methods

### Data Source

We obtained data from the year 2010 to 2014 from NIS, which is the largest publicly available all-payer inpatient care database in the United States (11). It is developed as a part of the Healthcare Cost and Utilization Project (HCUP) and is sponsored by the Agency for Healthcare Research and Quality. The NIS dataset includes data from all non-federal, short-term, general, and other specialty hospitals in the United States (excluding rehabilitation and long-term acute care hospitals) in the form of de-identified patient information containing demographics, discharge diagnoses, comorbidities, procedures, outcomes, and hospitalization costs. All the states participating in HCUP provide data to the NIS, covering >95% of the U.S. population. The database was designed to include data from a 20% sample of discharges from all participating hospitals. This large sample size of the NIS reduces the margin of error for estimates and delivers more stable and precise estimations (11). The study was exempt from an Institutional Review Board approval because HCUP-NIS is a publicly available database containing only de-identified patient information.

### Study Population

We used the International Classification of Diseases-Ninth Edition-Clinical Modification (ICD-9-CM) diagnosis codes 290.3, 290.41, 293.0, 293.1, 348.31, and 780 to identify all patients who are 65 years of age or older, hospitalized with a primary or secondary diagnosis of delirium. We then used ICD-9-CM diagnosis code 411.89 to identify patients with T2MI or demand ischemia. We excluded patients who were below the age of 65, and had a diagnosis of acute myocardial infarction (MI) or cardiogenic shock. Also, in order to reduce the possibility of data duplication, patients with an indicator of transfer to another acute care facility were excluded.

### Outcomes Measured

We studied the impact of demand ischemia in older patients with delirium during inpatient hospitalization. All-cause in-hospital mortality, which was defined as “died” during the hospitalization encounter in the NIS database, was the primary outcome of interest and median length of stay (LOS) was used as the secondary outcome.

### Patient Characteristics

Baseline patient characteristics included demographics (age, sex, race/ethnicity), and other clinically relevant characteristics (non-cardiogenic shock, arrhythmias, mechanical ventilation, post procedure bleeding, sepsis, acute kidney injury, fluid and electrolyte disorders, hypertension, dyslipidemia, smoking). Clinical Classification Software codes were used to identify the comorbidities described above.

### Statistical Analysis

A descriptive analysis was conducted to summarize variables for patients with demand ischemia and those without the condition, separately. The two groups of patients were compared with respect to each variable using a Fisher's exact test. A multivariate logistic regression model was used to associate DI with in-hospital mortality adjusting for all demographic and clinically relevant characteristics. Subgroup analysis was performed excluding critically ill patients with non-cardiogenic shock or mechanical ventilation. Length of stay was analyzed in the same manner restricted to survivors using a Poisson regression model. *p*-values smaller than 5% were considered statistically significant. All the statistical analyses were carried out in R 4.1.0.

## Results

### Baseline Characteristics

We included a total of 232,137 admission records from patients older than 65 years of age, who had delirium during the inpatient hospitalization from the year 2010 to 2014 NIS dataset. Out of these, 1,079 (0.49%) patients denoted the diagnosis of demand ischemia. Patients with demand ischemia were slightly older as compared to those without demand ischemia (79.43 ±8.20 vs. 78.16 ± 8.03 years, *p* < 0.001), as shown in Table 1. The incidence of demand ischemia increased significantly with aging as evident by a higher incidence in the >80-year-old age groups than that in the younger groups. The frequency of coded diagnoses of demand ischemia increased gradually with each year from 0.13% in 2010 to 0.90% in 2014. Patients with demand ischemia were associated with higher incidence of non-cardiogenic shock (18 vs. 4%, *p* < *0.0*01), mechanical ventilation (19 vs. 6%, *p* < *0.0*01), sepsis (37 vs. 14%, *p* < *0.0*01), acute kidney injury (41 vs. 24%, *p* < *0.0*01), chronic pulmonary disease (28.6 vs. 24.7%, *p* = 0.003), gastrointestinal bleed (2.4 vs. 1%, *p* < *0.0*01), and peripheral vascular disease (13.7 vs. 9.2%, *p* < 0.001) (Table 1).

**Table 1:** Baseline demographics and comorbidities of patients with and without demand ischemia.

**Variable**	**Delirium patients**	**Delirium patients**	***P*-value**
	**without demand**	**with demand**	
	**ischemia**	**ischemia**	
	**(*n* = 231,058)**	**(*n* = 1,079)**	
Died	27,155 (12%)	298 (28%)	<0.001
Age	78.16 ± 8.03	79.43 ± 8.20	<0.001
**Race**			0.049
Black	24,245 (10.49%)	105 (9.73%)	
Caucasian	165,466 (71.61%)	766 (70.99%)	
Hispanic	14,648 (6.34%)	64 (5.93%)	
Native American	1,119 (0.48%)	4 (0.37%)	
Other	20,798 (9.00%)	103 (9.55%)	
Black	24,245 (10.49%)	105 (9.73%)	
Non-cardiogenic shock	10,194 (4%)	196 (18%)	<0.001
Mechanical ventilation	14,409 (6%)	210 (19%)	<0.001
Ventricular arrythmia	2,666 (1.2%)	50 (4.6%)	<0.001
Sepsis	31,317 (14%)	396 (37%)	<0.001
SVT	43,023 (19%)	352 (33%)	<0.001
AKI	55,716 (24%)	442 (41%)	<0.001
Fluid and electrolyte disturbance	93,104 (40.3%)	590 (54.7%)	<0.001
Diabetes	64,748 (28.0%)	281 (26.0%)	0.158
Hypertension	123,249 (53.34%)	535 (49.58%)	0.015
Hyperlipidemia	87,752 (37.98%)	419 (38.83%)	0.586
Acute GI bleed	2,224 (1.0%)	26 (2.4%)	<0.001
Dementia	29,706 (12.9%)	127 (11.8%)	0.309
PAD	21,153 (9.2%)	148 (13.7%)	<0.001
CPD	57,185 (24.7%)	309 (28.6%)	0.004
Liver disease	9,422 (4.1%)	77 (7.1%)	<0.001
Malignancy	29,151 (12.6%)	155 (14.4%)	0.093
Age categories^*^			<0.001
(65, 70)	43,123 (99.6%)	173 (0.4%)	
(70, 75)	41,944 (99.59%)	171 (0.41%)	
(75, 80)	43,619 (99.62%)	166 (0.38%)	
(80, 85)	42,269 (99.51%)	208 (0.49%)	
(85, 90)	35,359 (99.44%)	200 (0.56%)	
≥ 90	24,744 (99.35%)	161 (0.65%)	
Year^*^			<0.001
2010	41,879 (99.87%)	53 (0.13%)	
2011	48,166 (99.77%)	113 (0.23%)	
2012	45,235 (99.59%)	185 (0.41%)	
2013	46,274 (99.40%)	280 (0.60%)	
2014	49,504 (99.10%)	448 (0.90%)	

* *Row-wise percentages*.

*Comparisons were made by using Fisher's exact test*.

*SVT, supraventricular arrhythmia: AKI, acute kidney injury; acute GI bleed, acute gastrointestinal bleed; PAD, peripheral artery disease; CPD, chronic pulmonary disease*.

### In-hospital Mortality

The overall mortality of patients with delirium was 12%. Patients with demand ischemia were noted to have higher unadjusted in-hospital mortality than those without demand ischemia (28 vs. 12%, *p* < 0.001), as shown in Table 1. However, there was no significant association between demand ischemia and in-hospital mortality after adjusting for demographics and other clinical variables (Table 2; adjusted OR: 1.14; 95% CI: 0.96–1.35; *p* = 0.14). As a significant number of deaths were from critically ill patients with either non-cardiogenic shock or mechanical ventilation (11,552 of 19,879, 58%), further analysis to exclude the aforementioned critically ill patients was performed. When patients with either non-cardiogenic shock or mechanical ventilation were excluded, demand ischemia was noted to have a significant association with in-hospital mortality (Table 3; adjusted OR: 1.39; 95% CI: 1.13–1.71; *p* = 0.002).

**Table 2:** Association between demand ischemia and in hospital mortality adjusting for demographics and clinically relevant characteristics.

**Characteristics**	**Adjusted odds**	**95% confidence**	***P*-value**
	**ratio (aOR)**	**interval**	
**Age categories**			
65–70	–	–	–
71–75	1.25	1.18–1.32	<0.001
76–80	1.59	1.50–1.67	<0.001
81–85	2.12	2.01–2.23	<0.001
86–90	2.77	2.63–2.93	<0.001
> 91	3.81	3.59–4.03	<0.001
**Race**			
Caucasian	–	–	–
Black	0.87	0.83–0.92	<0.001
Hispanic	0.70	0.66–0.75	<0.001
Native American	0.90	0.72–1.13	0.358
Asian or Pacific Islander	0.74	0.66–0.82	<0.001
Other	1.03	0.98–1.09	0.221
Demand ischemia	1.14	0.96–1.35	0.141
Female	0.91	0.88–0.94	<0.001
Non-cardiogenic shock	3.46	3.27–3.66	<0.001
Mechanical ventilation	11.81	11.29–12.35	<0.001
Ventricular arrythmia	2.07	1.86–2.30	<0.001
Sepsis	2.81	2.70–2.92	<0.001
SVT	1.47	1.42–1.52	<0.001
AKI	1.51	1.46–1.56	<0.001
Fluid and electrolyte disturbance	1.20	1.16–1.24	<0.001
Diabetes	0.88	0.85–0.91	<0.001
Hypertension	0.76	0.74–0.78	<0.001
Hyperlipidemia	0.64	0.62–0.66	<0.001
Acute GI bleed	1.11	0.96–1.28	0.151
Dementia	0.97	0.93–1.02	0.212
PAD	1.14	1.09–1.20	<0.001
CPD	1.10	1.07–1.14	<0.001
Liver disease	2.33	2.19–2.48	<0.001
Malignancy	2.97	2.86–3.09	<0.001

*aOR, adjusted odds ratio; CI, confidence interval; SVT, supraventricular arrhythmia; AKI, acute kidney injury; acute GI bleed, acute gastrointestinal bleed; PAD, peripheral artery disease; CPD, chronic pulmonary disease*.

**Table 3:** Association Between demand ischemia and in hospital mortality adjusting for demographics and clinically relevant characteristics in records without either non-cardiogenic shock or mechanical ventilation.

**Characteristic**	**Adjusted odds**	**95% confidence**	***P*-value**
	**ratio (aOR)**	**interval**	
**Age categories**			
(65, 70)	–	–	–
(71, 75)	1.12	1.05–1.20	0.001
(76, 80)	1.37	1.29–1.46	<0.001
(81, 85)	1.79	1.68–1.91	<0.001
(86, 90)	2.38	2.23–2.53	<0.001
≥91	3.42	3.20–3.64	<0.001
**Race**			
Caucasian	–	–	–
Black	0.79	0.75–0.84	<0.001
Hispanic	0.69	0.63–0.75	<0.001
Native American	0.97	0.75–1.25	0.827
Asian or Pacific islander	0.79	0.69–0.89	<0.001
Other	1.09	1.03–1.15	<0.001
Demand ischemia	1.39	1.13–1.71	0.002
Female	0.87	0.84–0.90	<0.001
Ventricular arrythmia	1.95	1.70–2.23	<0.001
Sepsis	3.13	3.00–3.26	<0.001
SVT	1.51	1.45–1.57	<0.001
AKI	1.58	1.52–1.64	<0.001
Fluid and electrolyte disturbance	1.27	1.22–1.31	<0.001
Diabetes	0.86	0.82–0.89	<0.001
Hypertension	0.77	0.74–0.80	<0.001
Hyperlipidemia	0.60	0.58–0.63	<0.001
Acute GI bleed	1.20	1.02–1.41	<0.001
Dementia	1.00	0.95–1.05	0.871
PAD	1.10	1.04–1.17	<0.001
CPD	1.21	1.16–1.25	<0.001
Liver disease	2.37	2.21–2.55	<0.001
Malignancy	3.32	3.19–3.46	<0.001

*aOR, adjusted odds ratio; CI, confidence interval; SVT, supraventricular arrhythmia; AKI, acute kidney injury; acute GI bleed, acute gastrointestinal bleed; PAD, peripheral artery disease; CPD, chronic pulmonary disease*.

### Length of Stay

Among the survivors excluding those with non-cardiogenic shock or mechanical ventilation, patients with demand ischemia (median 4 days, range: 0–85) had a longer median LOS than those without demand ischemia (median 4 days, range: 0–354) (*p* < 0.001) but the LOS rate ratio (RR) comparing demand ischemia patients to non-demand ischemia patients after adjusting for demographic and clinically relevant characteristics was not statistically significant (adjusted *RR* 1.03; 95% *CI*: 1.00–1.06; *p* = 0.096) (Table 4).

**Table 4:** Association between demand ischemia and length of stay in survivors without either non-cardiogenic shock or mechanical ventilation adjusting for demographics and clinically relevant characteristics.

**Characteristics**	**Adjusted rate**	**95% confidence**	***P*-value**
	**ratio (aRR)**	**interval**	
**Age categories**			
(65, 70)	–	–	<0.01
(71, 75)	0.97	0.96–0.97	<0.01
(76–80)	0.95	0.94–0.95	<0.01
(81–85)	0.90	0.89–0.91	<0.01
(86–90)	0.87	0.86–0.87	<0.01
≥91	0.82	0.81–0.82	<0.01
**Race**			
Demand ischemia	1.03	1.00–1.06	0.096
Female	0.95	0.95 0 0.95	<0.01
**Race**			
Caucasian	–	–	<0.01
Black	1.1	1.09–1.11	<0.01
Hispanic	1.06	1.05–1.07	<0.01
Native American	0.96	0.93–0.99	<0.01
Asian or Pacific islander	1.09	1.07–1.10	<0.01
Other	1.03	1.02–1.04	<0.01
SVT	1.14	1.14–1.16	1.15
AKI	1.1	1.10–1.11	1.11
Ventricular arrythmia	1.36	1.34–1.39	1.39
Sepsis	1.18	1.18–1.19	1.19
Hypertension	0.97	0.97–0.98	0.98
Hyperlipidemia	0.96	0.96–0.96	0.96
Fluid and Electrolyte disturbance	1.09	1.09–1.10	1.1
Diabetes	0.95	0.95–0.96	0.96
PAD	1.1	1.09–1.11	1.11

*SVT, supra ventricular tachycardia; AKI, acute kidney injury; PAD, peripheral artery disease*.

## Discussion

The results of our study demonstrated that in older patients with delirium, demand ischemia did not have a significant impact on mortality in critically ill patients, such as those with non-cardiogenic shock or respiratory failure requiring mechanical ventilation. However, excluding patients with the aforementioned two conditions, demand ischemia was associated with significantly higher in-hospital mortality, implicating its value as an independent predictor of mortality.

The number of patients diagnosed with demand ischemia increased gradually from the year 2010 to 2014 in the NIS database. This might be attributed to improved definition, more precise profiling, and increased awareness of this condition among clinicians (12). Demand ischemia also became more frequent with aging and was highest in the age group older than 90 years. Increased prevalence and severity of CAD with aging resulting in lower thresholds for invoking demand ischemia may explain the increasing trend of demand ischemia with aging.

The reported in-hospital mortality rates with septic shock and demand ischemia range from 26.9 to 56% (13–17). The mortality rate in our cohort of critically sick patients with non-cardiogenic shock or respiratory failure requiring mechanical ventilation was 59.2%. This slightly higher mortality rate in our study was likely due to older age, a sicker population, and the presence of delirium. Our results, nevertheless, are consistent with prior studies showing no impact of non-coronary troponin elevation on mortality in patients with septic shock (13, 14).

However, overall data related to the impact of demand ischemia on mortality in critically sick patients are conflicting, with some of the studies showing higher in-hospital mortality (15, 16).

Demand ischemia remained an independent predictor of mortality in delirium patients without non-cardiogenic shock or mechanical ventilation. Previous studies evaluating the impact of delirium in older patients with acute myocardial infarction have reported in-hospital mortality ranging from 10.5% in all comers to 42.7% in patients with ST-elevation MI (9, 10). In-hospital mortality with demand ischemia ranges from 12 to 26.9% and is driven primarily by acute systemic illness and comorbidities (13, 18, 19). The overall mortality with demand ischemia in our study was 28 and 15.5% in patients without mechanical ventilation and/or non-cardiogenic shock. Our results are consistent with the previous analysis, even though we included older patients with concurrent delirium.

Demand ischemia and delirium are highly prevalent in older hospitalized adults. Both of these entities often have shared predisposing and precipitating factors, complex multifactorial etiology, and are associated with poor outcomes (20). The interplay between the two is not well understood; therefore, prevention, timely diagnosis and management are necessary to reduce short- and long-term poor outcomes. Once delirium is diagnosed, it is necessary to identify and treat underlying triggers to reduce downstream complications. Once a myocardial injury is detected, the first step is to determine if the myocardial injury is due to a plaque disruption (type I MI) or supply demand mismatch (type II MI). Timely differentiation of type I and type II MI is often challenging, however, critical for optimal care, as the treatment widely differs (21). A combination of symptoms, electrocardiogram (EKG) changes, cardiac biomarkers, invasive and non-invasive imaging can be utilized to guide further management. However, the presence of delirium adds complexity to assessment and decision-making as patients may have marked inattention and/or behavioral disturbances that may preclude appropriate cardiac evaluation. If based on symptoms, EKG changes, pre-test probability of type I MI is low, and troponin elevation occurs in the context of acute illness such as infection, anemia, respiratory failure, and arrhythmias, and multiple comorbidities, type II MI is likely, even though acute plaque ruptures can be triggered by systemic inflammation in the setting of acute illness (22). In cases of diagnostic uncertainty, coronary angiography or non-invasive imaging modalities such as coronary CTA, echocardiography and cardiac MRI may be more suitable to aid in the differentiation of type I and type II MI (23).

Treatment strategies for type II MI are directed at the specific underlying etiology. Demand ischemia is associated with higher short- and long-term all-cause mortality as well as cardiovascular mortality (18, 21, 24–26). The higher early mortality is driven primarily by non-cardiovascular acute illness. There are no treatment options to reduce in-hospital mortality. Beta-blockers can be used in hemodynamically stable patients to reduce the heart rate and subsequent myocardial oxygen demand. However, whether it reduces mortality in the short term is not known. Long-term mortality is driven by both non-cardiovascular and cardiovascular causes, which emphasizes the importance of cardiac evaluation to improve long term outcomes (26, 27). Once patients are discharged from the hospital, they may benefit from a further cardiac work up for underlying cardiovascular disease such as atherosclerosis, structural heart disease or heart failure (23).

However, following discharge, delirium may be protracted, and is strongly associated with several short- and long-term complications such as increased risk of institutionalization, falls, persistent cognitive dysfunction, poor quality of life, long term disability, and mortality (5, 20, 28, 29). Therefore, further cardiac work up may not be feasible or desired depending upon frailty, functional status, cognitive impairment, the likelihood of recovery, severity of psychiatric and non-cardiac comorbidities, polypharmacy, and most importantly patient's preference and goals of care. And often, depending upon a patient's preference, treatments aimed at improving function and reducing symptoms may be more suitable than those focused on prolonging survival (30).

Our study has certain limitations. First, because NIS is an administrative database, the accuracy of the data depends highly on the training and expertise of the coders. Hence, there is the potential of unrecognized miscoding of diagnosis codes, leading to underestimation of demand ischemia or delirium based on ICD-9-CM coding (31). Second, we were not able to determine the time of onset, duration, type or severity of delirium as well as demand ischemia occurring during the hospitalization. We were also unable to examine the temporal relation between demand ischemia and the time of occurrence of delirium. Third, all cases coded as T2MI may not only be due to demand supply mismatch, and a plaque rupture or erosion cannot be completely ruled out without a coronary angiogram. Fourth, data on laboratory values, use of adjunctive medications and cause of death were not available. Fifth, this is a retrospective observational study and an unobserved confound might have occurred, even after accounting for a relatively large number of covariates. Lastly, data in the NIS are limited to in-hospital events, and information on long-term outcomes is not available.

Despite the limitations, our study has significant value. First, we used a large dataset that is representative of a large national population, leading to a higher power as well as generalizability. Second, our results emphasize the utmost need for finding appropriate novel diagnostic and intervention strategies for delirium as well as demand ischemia to reduce short- and long-term poor outcomes. In critically sick delirious patients with non-cardiogenic shock or mechanical ventilation, demand ischemia appears to be an innocent bystander without significant impact on in-hospital mortality. Given a >55% mortality rate in these patients, depending upon clinical circumstances, goals of care, including end-of life discussions may be appropriate. Patients with delirium who are not critically sick may benefit from further cardiac intervention after an individualized risk-benefit assessment (23).

## Conclusion

Delirium and demand ischemia is common in hospitalized older individuals and represents end organ damage as a result of acute illness. Demand ischemia did not affect mortality in critically ill patients, where aggressive cardiac intervention may not be warranted. In other non-critical patients. However, demand ischemia was significantly associated with mortality after adjusting for demographics and clinical characteristics, likely paralleling severity of the underlying acute illness. Measures aimed at the prevention and management delirium and demand ischemia are of paramount importance.

## Contributions

Troponin assays are frequently used to diagnose myocardial injury, and are one of the cornerstones for the diagnosis of acute coronary syndrome (ACS). Troponin assays, however, are not specific and can occur due to non-coronary causes as well. This is termed as demand ischemia and occurs due to myocardial supply demand mismatch. Delirium is another such condition that occurs due to demand supply mismatch in brain. Delirium often shares the similar precipitating factors and pathophysiological mechanisms. Our results indicate that demand ischemia did not have a significant impact on mortality in critically ill older patients with delirium. However, in patients who are not critically sick, demand ischemia was associated with significant higher in-hospital mortality, implicating its value as an independent predictor. These results have important clinical implications, such as, in critically sick patients with delirium and demand ischemia, aggressive cardiac intervention may not be warranted. And perhaps, goals of care, including end-of life discussions may be appropriate as per the clinical condition given >50% mortality in these patients. Patients with delirium who are not critically sick, may benefit from further cardiac intervention after an individualized risk-benefit assessment.

## Data Availability Statement

The original contributions presented in the study are included in the article/supplementary material, further inquiries can be directed to the corresponding author.

## Ethics Statement

Ethical review and approval was not required for the study on human participants in accordance with the local legislation and institutional requirements. Written informed consent for participation was not required for this study in accordance with the national legislation and the institutional requirements.

## Author Contributions

MK: conceptualization, writing, editing, and reviewing. SP, GK, and KC: conceptualization, editing, and reviewing. LG, CK, and HS: statistical analysis, editing, and reviewing. All authors contributed to the article and approved the submitted version.

## Conflict of Interest

The authors declare that the research was conducted in the absence of any commercial or financial relationships that could be construed as a potential conflict of interest.

## Publisher's Note

All claims expressed in this article are solely those of the authors and do not necessarily represent those of their affiliated organizations, or those of the publisher, the editors and the reviewers. Any product that may be evaluated in this article, or claim that may be made by its manufacturer, is not guaranteed or endorsed by the publisher.
